# Chemical and Microbial Quality of Groundwater in Siloam Village, Implications to Human Health and Sources of Contamination

**DOI:** 10.3390/ijerph15020317

**Published:** 2018-02-12

**Authors:** John Ogony Odiyo, Rachel Makungo

**Affiliations:** Department of Hydrology and Water Resources, University of Venda, Thohoyandou 0950, South Africa; rachel.makungo@univen.ac.za

**Keywords:** ANOVA, faecal contamination, fluorides, groundwater quality, health risks, nitrates

## Abstract

Due to inaccessibility of potable water, rural communities drill boreholes within their homesteads despite vulnerability to groundwater contamination and associated health risks. This study assessed the quality of groundwater, identified potential sources of contamination and potential human health risks in Siloam Village, South Africa. Statistical difference between similar water quality parameters at different sites was determined at a significance level (α) of 0.05. Water quality parameters with serious potential health effects on human beings were correlated with selected water quality parameters to understand the nature of correlation and possible sources of contamination. Fluorides and nitrates had excessively high concentrations associated with tooth damage and pronounced skeletal fluorosis, and methaemoglobinaemia in infants and mucous membrane irritation in adults, respectively. There were statistically significant differences between means of most water quality parameters. Contrasting correlation of fluoride with calcium and pH indicated the need to further identify local sources and fluoride control mechanisms. Correlation of nitrate with chloride mostly indicated that faecal contamination is the potential source of high nitrates in groundwater. This requires further verification. Presence of total coliforms and *E. coli* in most boreholes indicated potential presence of faecal contamination. The need to educate borehole owners’ on possible strategies to minimise groundwater pollution was identified.

## 1. Introduction

Composition of precipitation, anthropogenic activities, geological structure and processes within an aquifer determine the quality of groundwater [1]. Dissolution of minerals within the soil, sediment, and bedrock contribute to different cations and anions in groundwater depending on the geology of an area. For example, natural geology has been found to contribute to high fluoride, calcium, and trace metal concentrations in Kwazulu Natal [2], water hardness in the Central Free State [3], fluoride concentrations in Limpopo, Northern Cape, North-West and KwaZulu-Natal Provinces [4], in South Africa. Dedzo et al. [5] and Basavarajappa et al. [6] associated weathering of rock minerals with groundwater chemistry in Central-East Cameroon and Kanata, India, respectively.

Poor and deteriorating groundwater quality associated with diverse anthropogenic sources is widespread in South Africa [7]. Faecal matter from pit latrines also has potential to contaminate groundwater, thereby threatening human health [8]. Despite the water quality problems associated with groundwater, most rural areas of South Africa located in semi-arid remote areas characterized by inadequate potable water supply are use groundwater for domestic purposes. Due to lack of access to potable water, residents in rural areas drill their own private boreholes without considering the potential sources of groundwater contamination. In these areas, boreholes are drilled within the vicinity of pit latrines and subsistence farms within the owners’ homesteads.

Holland [9] confirmed microbial contamination of numerous village water supply boreholes that are sited close to pit latrines in basement areas of Limpopo Province. In the Tshitale-Hlanganani region of Limpopo Province boreholes drilled close to sanitary facilities (pit latrine and septic tanks) had high microbial contamination [10]. Similar cases have been noted in rural areas of Zimbabwe [11], South West Nigeria [12], Bihar State in India [13], amongst others. Dissolution of rocks and infiltration from pit-latrines were identified as sources of groundwater pollution in Kwale District, Kenya [14]. In Siloam Village, agricultural practices and washing of clothes within the vicinity of boreholes were associated with high concentrations of calcium, magnesium and nitrate in groundwater [15]. The current study is focused on determining the quality status of groundwater from private boreholes and implications for domestic use, as well as inferring possible sources of contamination. This will be used to inform private borehole owners of the quality status of their borehole water and suggest ways of minimising groundwater contamination. The study also builds up on previous studies and provide additional information on water quality status of Siloam Village which will aid in generating solutions and decision making. Odiyo and Makungo [16] determined that calcium and pH influence fluoride concentrations in groundwater in Siloam Village but did not determine the relationship between these parameters. The latter study associated low calcium concentration to the high concentration of fluoride, while increased fluoride concentrations were attributed to alkaline pH in some of the boreholes in Siloam Village. Correlation can assist in identifying the sources of pollution and the significance of the water quality parameters. This is crucial when developing strategies to minimise pollution. Correlation studies also aid in choosing parsimonious parameters for frequent determination of water quality status [17], since it is cumbersome and costly to regularly monitor all water quality parameters. Thus, this will aid in ensuring continuous monitoring of the quality of groundwater.

## 2. Materials and Methods

### 2.1. Study Area

**Figure 1:**
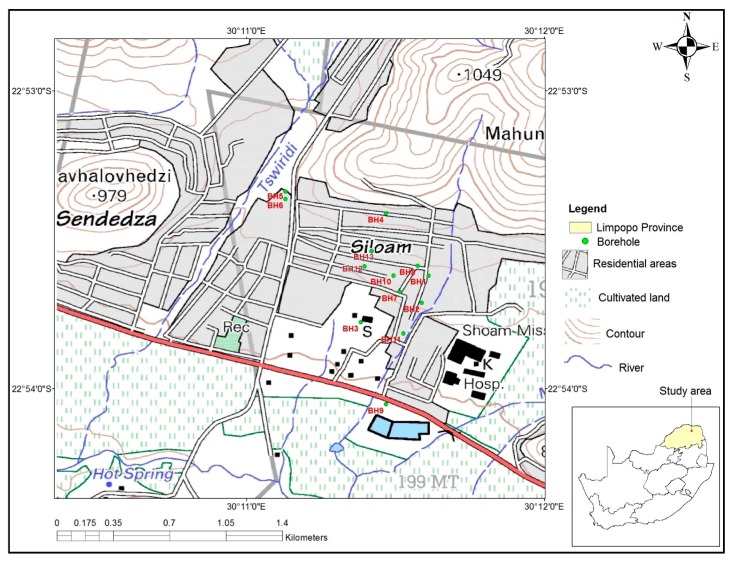
Location of the study area.

Siloam Village (Figure 1) falls within the semi-arid Nzhelele area in Limpopo Province, South Africa. Nzhelele River is the main river with low and variable flow pattern due to low and unpredictable rainfall (average 350 to 400 mm) within its catchment [18]. The area is characterized by basic extrusive basalt rocks of the Sibasa formation, Soutpansberg Group. Groundwater occurs in intergranular and fractured rocks with borehole yields ranging from 0.5 to 2 L/s [19]. Within Nzhelele area, boreholes depths varies from 65 to 85 m while average depth to groundwater is 15–25 m [20]. The use of groundwater to supplement surface water and harvested rainwater supply is common in Siloam Village. In addition to one public borehole that exists in Siloam Village, most residents have drilled boreholes within their homesteads to supplement their water needs.

### 2.2. Methods

Groundwater samples from 11 boreholes in Siloam Village (BH1 to BH11 in Figure 1) were collected in sterile 1 L plastic containers and transported to the University of Venda research laboratory for physico-chemical analysis. The samples were collected once a month from August 2013 to January 2014. August and September 2013 fall in the dry season, while October to December 2013 and January 2014 are in the wet (rainfall) season. EC and pH were measured using Cyberscan PC510 benchtop meter while turbidity was measured using Eutech TN 100 turbidity meter. Samples were kept at 4–10 °C during transport to the laboratory. Non-metals (nitrates, fluoride, chloride, phosphate, sulphate) and metals (copper, manganese, zinc, calcium, potassium, magnesium, and iron) were analysed using 850 Professional Ion Chromatography (Anions Cation Metrohm Carbon dioxide Suppression (AnCat MCS) model, Metrohm (pty) Ltd., Pretoria, South Africa) and atomic absorption spectroscopy, respectively. These non-metallic and metallic parameters were selected because they are general indicators of water quality, which may lead to health problems [21], if they are in excessive concentrations. All equipment were calibrated following manufacturers’ instructions and the samples were analysed in triplicate for quality assurance purposes. Samples were analysed within 24 h of collection. This is below the seven day period reported by the Department of Water Affairs and Forestry (DWAF) [22] for samples collected for chemical analysis, provided that they are kept in a cool environment.

Descriptive statistics (minimum, mean, maximum) were calculated to summarise the results of physico-chemical parameters and to assess the overall quality status of groundwater in the study area. The results were also compared with DWAF [23] target water quality ranges (TWQRs) for domestic use to infer the potential health risks. TWQR gives concentration range that have no effects on human health for a given water quality parameter. Water quality parameters with serious potential health effects on human beings were correlated with selected water quality parameters to understand the nature of correlation and possible sources of contamination. Relationships between fluoride with calcium and pH were determined from correlation coefficient at significance level (α) of 0.05. This was aimed at establishing the control mechanism of fluoride in groundwater, and nature of correlation and significance of quality parameters on fluoride as suggested by [24,25]. Nitrates were also correlated with chloride and potassium to identify and determine potential sources of nitrate contamination. Statistical differences between similar water quality parameters at different sites was determined using single factor analysis of variance (ANOVA) within Microsoft Excel (2013, Microsoft, Johannesburg, South Africa). The tests were run at a significance level (α) of 0.05.

Microbial quality indicators (total coliform, heterotrophic bacteria, *Escherichia coli* (*E. coli*) and faecal coliform were assessed in a previous study conducted by Mudau [26]. These were used to assess microbial status of groundwater and verify potential presence of faecal contamination of groundwater as indicated by the correlation between nitrates and chlorides. Total coliform and heterotrophic bacteria informed hygienic quality and general microbial quality of water while *E. coli* and faecal coliform indicated potential faecal contamination of groundwater.

## 3. Results

### 3.1. Groundwater Quality and Implications to Human Health

Descriptive statistics for water quality parameters for a six month period are in Table 1 and Table 2. pH was mostly within TWQR of 6–9 except for maximum values for August, November, and December 2013, and January 2014, which were slightly above 9. Maximum and mean turbidity values for August 2013 and January 2014 exceeded TWQR of 0–1 NTU indicating chances of transmission of diseases by micro-organisms associated with particulate matter. Maximum and mean EC values were above TWQR of 070 mS/m for the entire period. Water is noticeably salty but is well tolerated and no health effects are likely to occur at EC values from 70 to 150 mS/m [23]. Water with EC values from 150 to 300 mS/m is associated with marked, salty taste, though it has no adverse health effects in the short term [23]. Maximum EC values for September, October, and December 2013 were only slightly above 150 mS/m and, therefore, may not pose serious aesthetic and health risks.

Minimum, mean, and maximum fluoride concentrations were above TWQR of 0–1 mg/L except for minimum concentration for August 2013 (Table 1). Minimum and mean fluoride concentrations mostly ranged from 1 to 5 mg/L which is associated with slight mottling of dental enamel in sensitive individuals [23]. Maximum fluoride concentrations for September to November 2013, and January 2014, and mean concentrations for October to November 2013 and January 2014 were within the range of 4 to 6 mg/L which can severely damage the teeth particularly those of infants and soften the enamel and dentine if water is used continuously [23]. Maximum fluoride concentrations for August and December 2013 were within the range of 6 to 8 mg/L, which can severely damage teeth and cause pronounced skeletal fluorosis on long-term exposure [23].

Mean chloride concentrations for August and December 2013, and January 2014, and maximum for November 2013 exceeded DWAF [23] TWQR of 0 to 100 mg/L (Table 1), though they had no aesthetic or health effects. Maximum chloride concentrations for August to October 2013, December 2013 and January 2014 exceeded 200 mg/L. Water with chloride concentrations exceeding 200 mg/L has a distinctly salty taste, but no health effects [23]. Mean and maximum nitrates were >20 mg/L and, hence, exceeded TWQR of 0 to 6 mg/L. Nitrate concentrations >20 mg/L are associated with methaemoglobinaemia in infants and occurrence of mucous membrane irritation in adults [23]. Maximum sulphates for October and November 2013, and January 2014 exceeded a TWQR of 200 mg/L. DWAF [23] indicated that water with sulphates exceeding 200 mg/L can cause diarrhoea and it is salty and bitter. Most metals were within their specific TWQR (Table 2), except for maximum and mean manganese concentrations for September 2013 to January 2014, and September and December 2013, respectively, which exceeded the TWQR of 0 to 0.05 mg/L. Water with manganese concentrations from 0.05 to 0.1 mg/L is within tolerable range associated with no health effects [23]. Concentrations of Mn between 0.15 to 1.0 and 1.0 to 2.0 mg/L indicate thresholds for significant staining and very severe staining, respectively, though the water has no health effects [23]. Thus, maximum manganese concentrations in September and December 2013, and January 2014, which were >0.15, but <1 mg/L could cause significant staining, but no health effects. Maximum iron concentrations in December 2013 and January 2014 ranged from of 0.1 to 0.3 mg/L which, has very slight effects on taste, but no health effects [23].

### 3.2. ANOVA and Correlations

Results of ANOVA test (Table 3) showed statistically significant difference between means of most of the water quality parameters since *p*-values were less than 0.05, except for chloride, copper and zinc. Misi [27] reported no significant differences (*p* > 0.05) between mean values of pH, turbidity, EC, chloride, fluoride, iron, zinc, and copper across sampling sites located in basement aquifer of Upper Manyame Sub-catchment in Zimbabwe. This was explained to be due to the fact that sampling was conducted in the rainy season, hence, less variation could be expected due to the dilution effect from the rain. Sampling period in the current study partially covered both dry and rainy seasons showing that variations in rainfall possibly resulted in significant differences between means of most of the water quality parameters. Mean values for chloride, copper and zinc were, however, not affected by seasonal variations in rainfall since their *p*-values were higher than 0.05.

**Figure 2:**
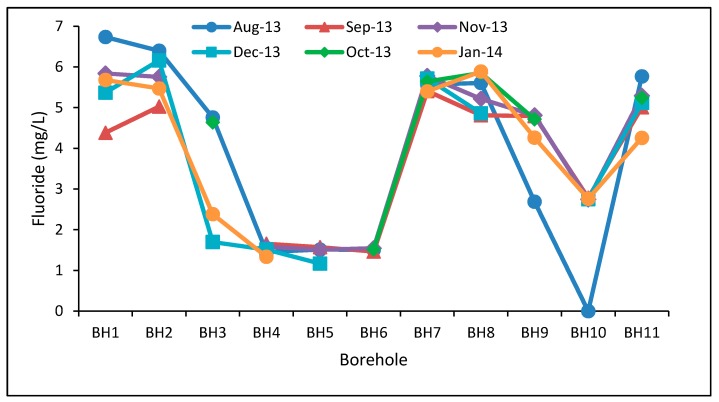
Variations of fluoride concentrations.

Nitrates and fluorides were the only parameters with excessively high concentrations in groundwater which are associated with health effects on human beings, and their correlation with related parameters was determined. Negative correlation between calcium and fluoride was found in seven of the boreholes while four had positive correlation (Table 4). The contrasting correlations is an indication of the local variations of geological formations that are in contact with groundwater within the aquifer. This has resulted to the spatial variations in fluoride concentrations in the study area (Figure 2). Though surface geology around all boreholes in the area is similar, it is possible that there are variations in geology in the deep formations where groundwater is found. Odiyo and Makungo [16] associated increase in calcium with decrease in fluoride in Siloam Village, thereby, suggesting negative correlation of the two variables. The results of the latter study were, however, limited to three sites (two boreholes and one hotspring) which are excluded in the current study. Negative correlation between fluoride and calcium is attributed to low solubility of fluoride from fluoride bearing rocks [28], suggesting the possibility of ion-exchange process [29].

Ncube [4] reported negative correlation of calcium and fluoride for a borehole in Stelnberg and positive correlation for boreholes in Tugela and Zalexbayi in South Africa. Negative correlations were associated with moderate fluoride concentrations (0.3–1.0 mg/L) while positive correlations were associated with low and high fluoride concentrations. The results of this study did not follow this trend. For example, BH2 with high fluoride concentrations (5.03–6.40 mg/L) and BH5 with low concentrations (1.17–1.57 mg/L) had negative correlation while BH4 with low fluoride concentrations (1.34–166 mg/L) and BH1 with high concentrations (4.38–6.74 mg/L) had a positive correlation (Table 4). Odiyo and Makungo [16] reported increased fluoride concentrations resulted from reduced dilution effect on groundwater chemical composition due to arid/dry conditions in Siloam Village. In addition, the fluorite, which is a fluoride bearing rock, was identified as the likely source of fluoride in Siloam Village in the latter study. This indicates that further investigations are required to identify the local sources and fluoride control mechanisms in the study area. Chakraborti [30] reported positive correlation of fluoride with calcium in groundwater in Assam, India, which [31] attributed to the presence of limestone in that area. Avtar et al. [32] indicated positive correlation between pH and calcium with groundwater in Chhatarpur area, Madhya Pradesh, India. The correlation between calcium and fluoride was linked to minerals like fluorspar, apatite, and fluorapatite. Liu et al. [33] noted that positive correlation between Ca and F indicated that they have the same origin.

Correlation between pH and F in five of the boreholes was positive, while the rest had negative correlation. A positive relationship with pH indicates towards a possible leaching of fluoride under high alkaline conditions of water [29]. Negative correlation between pH and F and positive correlation between Ca and F was reported in Iran [34]. Umarani and Ramu [35] reported that negative correlation of pH and F in groundwater from Tamilnadu, India, indicated though fluoride concentrations were low (0.02 to 1.54 mg/L), they could still dissolve in groundwater. Fluoride concentrations in the latter study were within. Nephalama and Muzerengi [36] also attributed negative correlation of pH and F to low levels of fluoride in the rock in Masisi Village in Limpopo Province, South Africa where fluoride concentrations ranged from 0.31 to 0.94 mg/L. In the current study, BH5, BH6 and BH10 had negative correlation of pH and F, and relatively low F concentrations which ranged from 1.17 to 1.57, 1.46 to 1.55, and 0.00 to 2.79 mg/L, respectively (Table 4 and Figure 2). Thus, these low concentrations are likely to indicate low fluoride concentrations in the rocks at these sites and, therefore, explains negative correlation between F and pH. These findings therefore show variable occurrence of fluoride bearing rocks in the study area.

BH1 and BH7 had relatively high positive correlations of potassium and nitrates (Table 5). Correlation between potassium and nitrates indicates that contamination is mainly from fertilizers [37]. BH3, BH4, BH6, BH7, BH8, and BH11 had positive high correlation of nitrates and chlorides (Table 5). Correlation between chlorides and nitrates indicates that contamination is mainly from animal and human wastes [37]. The boreholes are located within homesteads where there are no animals or livestock that can generate animal waste which has potential to pollute groundwater. Thus, the only likely source of groundwater contamination is faecal matter from pit latrines which are within the vicinity of the boreholes. However, further studies are required verify to if faecal matter is the source of nitrate contamination of groundwater in Siloam Village. The distances between pit latrines and boreholes in the study area as measured by Mukhumo [38] and presented in Table 5, show that boreholes are within proximity of pit latrines. The recommended minimum safe distance between point source of groundwater supply and pit latrine should be >50 m [39]. Thus, there is high risk of groundwater contamination by human wastes in Siloam Village. Mudau [26] showed that there is contamination of groundwater by faecal matter from pit latrines that are not placed at an appropriate distance from the boreholes in Nzhelele area including Siloam Village. Results from [11] indicated that pit latrines impacted on microbiological groundwater quality up to 25 m lateral distance. The ease through which aquifer material/geology restricts or permits movement of microbial contaminants into the aquifer influences the distance up to which microbial contamination of groundwater occurs. Bessong et al. [40] indicated that microbial contamination of groundwater was reduced due to local geological barriers within the aquifer and/or filtration through attachment to soil media.

Microbial water quality indicators obtained in boreholes BH12 and BH13 (Figure 1) in Table 6 mostly exceeded their specific TWQR. This indicated a high risk of infectious disease transmission [26]. Studies done in rural areas of South Africa (for example, Potgieter et al. [10], Mpenyana-Monyatsi et al. [41], Palamuleni and Akoth [42]) have also indicated that faecal contamination of groundwater poses health risks to communities. Faecal coliform and *E. coli* are indicators of faecal pollution of water and their presence in most of the boreholes, though determined within a different study period, therefore, support the high correlation of nitrates and chloride obtained in the current study.

## 4. Conclusions

The study showed that nitrates and fluorides were the only parameters with excessively high concentrations in groundwater which are associated with health effects on human beings. Potential risks associated with high concentrations of fluorides and nitrates include tooth damage and pronounced skeletal fluorosis on long-term exposure, and methaemoglobinaemia in infants and the occurrence of mucous membrane irritation in adults, respectively. The means of most of the water quality parameters were statistically different, as shown from the results of the ANOVA test.

Though earlier studies have identified potential sources of fluoride in Siloam Village, the contrasting correlations obtained with calcium and pH in this study indicate the need for further investigations to identify the local sources and fluoride control mechanisms in the study area. Microbial contamination of groundwater mainly from pit latrines was also identified. Subsistence farming was also found to contribute to nitrate pollution in some of the boreholes. There is a need to discuss possible strategies to minimise groundwater pollution with borehole owners in Siloam Village. These include relocation of pit latrines to acceptable distances from boreholes and efficient application of fertilizers to only the levels required by the crops.

## Figures and Tables

**Table 1:** Descriptive statistics for physical water quality parameters and non-metals.

Parameter	Descriptive Statistic	August 2013	September 2013	October 2013	November 2013	December 2013	January 2014	TWQR
pH	Min	7.14	7.27	7.22	7.00	7.26	6.83	6–9
	Max	**9.06**	8.88	8.86	**9.08**	**9.29**	**9.28**	
	Mean	7.68	7.87	7.75	7.83	7.88	7.86	
	STDEV	0.53	0.54	0.69	0.83	0.64	0.89	
Turbidity (NTU)	Min	0.00	0.00	0.01	0.00	0.00	0.00	1
	Max	**25.91**	0.89	0.55	0.47	0.69	**11.85**	
	Mean	**2.54**	0.17	0.18	0.09	0.19	**1.80**	
	STDEV	7.77	0.27	0.23	0.15	0.26	3.97	
EC (mS/m)	Min	34.90	36.60	43.80	36.10	23.68	30.10	70
	Max	**141.80**	**156.60**	**158.80**	**141.00**	**151.70**	**139.90**	
	Mean	81.96	90.83	86.02	84.68	85.63	90.33	
	STDEV	38.10	43.07	40.94	33.95	43.77	42.27	
Fluoride (mg/L)	Min	0.00	**1.40**	**1.52**	**1.51**	**1.17**	**1.34**	1
	Max	**6.74**	**5.41**	**5.85**	**5.84**	**6.16**	**5.89**	
	Mean	**3.82**	**3.69**	**4.60**	**4.01**	**3.82**	**4.16**	
	STDEV	2.41	1.64	1.59	1.92	2.01	1.64	
Chloride (mg/L)	Min	5.73	0.00	0.00	0.00	24.89	0.00	100
	Max	**1177.40**	**223.58**	**223.13**	**198.22**	**296.27**	**410.86**	
	Mean	**198.01**	83.40	99.61	87.84	**134.12**	**160.56**	
	STDEV	332.66	69.43	83.88	67.23	95.93	145.55	
	Min	0.22	6.77	10.10	2.66	6.10	7.92	
Sulphate (mg/L)	Max	188.36	64.18	**365.36**	**313.79**	174.58	**404.01**	200
	Mean	34.45	24.07	80.20	43.75	44.99	70.12	
	STDEV	53.14	20.33	140.29	95.30	54.39	126.57	
Nitrate (mg/L)	Min	0.00	0.00	0.00	0.00	0.00	0.00	6
	Max	**137.04**	**409.93**	**65.51**	**209.94**	**596.42**	**543.27**	
	Mean	**40.96**	**88.69**	**34.43**	**45.57**	**99.99**	**107.63**	
	STDEV	48.36	129.86	25.76	64.61	193.17	175.59	

Note: Values in bold exceeded TWQR.

**Table 2:** Descriptive statistics for metals.

Parameter	Descriptive Statistic	August 2013	September 2013	October 2013	November 2013	December 2013	January 2014	TWQR
Magnesium (mg/L)	Min	0.33	0.33	5.46	0.09	0.54	1.08	30
	Max	10.86	11.19	11.26	11.22	10.57	11.27	
	Mean	7.98	8.35	9.29	8.32	8.34	8.14	
	STDEV	3.64	3.82	2.07	3.61	3.20	3.96	
Manganese (mg/L)	Min	0.01	0.01	0.00	0.01	0.01	0.01	0.05
	Max	0.04	**0.56**	**0.10**	**0.14**	**0.27**	**0.23**	
	Mean	0.02	0.09	0.02	0.04	0.06	0.05	
	STDEV	0.01	0.17	0.04	0.05	0.09	0.07	
Potassium (mg/L)	Min	0.30	0.38	0.45	0.34	0.00	0.76	50
	Max	4.67	5.84	3.94	7.70	8.67	7.30	
	Mean	2.01	2.12	1.78	2.89	2.36	2.77	
	STDEV	1.48	1.95	1.27	2.70	2.96	2.04	
Copper (mg/L)	Min	0.00	0.00	0.01	0.01	0.00	0.01	1
	Max	0.02	0.02	0.09	0.04	0.01	0.21	
	Mean	0.01	0.00	0.02	0.02	0.01	0.03	
	STDEV	0.01	0.01	0.03	0.01	0.00	0.07	
Zinc (mg/L)	Min	0.00	0.00	0.01	0.00	0.00	0.01	3
	Max	0.05	0.30	0.84	0.37	1.00	0.88	
	Mean	0.01	0.06	0.16	0.08	0.15	0.22	
	STDEV	0.01	0.09	0.33	0.12	0.33	0.30	
Calcium (mg/L)	Min	0.03	0.00	0.05	0.07	0.00	0.02	32
	Max	4.12	1.89	5.25	4.67	3.26	5.59	
	Mean	0.78	0.38	1.40	1.44	0.77	1.35	
	STDEV	1.20	0.67	2.01	1.59	1.17	1.94	
Iron (mg/L)	Min	0.00	0.00	0.00	0.00	0.00	0.00	0.1
	Max	0.00	0.01	0.00	0.06	**0.14**	**0.17**	
	Mean	0.00	0.00	0.00	0.01	0.02	0.02	
	STDEV	0.00	0.00	0.00	0.02	0.05	0.06	

Note: Values in bold exceeded TWQR.

**Table 3:** Statistical significance based on single factor ANOVA.

Parameter	*p*-Value	Statistical Significance
pH	6.69444 × 10^−12^	Yes
EC	4.1007 × 10^−19^	Yes
Turbidity	3.03 × 10^−2^	Yes
Fluoride	7.98306 × 10^−15^	Yes
Chloride	0.5255362	No
Sulphate	6.17901 × 10^−10^	Yes
Nitrate	1.28 × 10^−4^	Yes
Manganese	6.46 × 10^−3^	Yes
Potassium	1.94 × 10^−7^	Yes
Copper	0.2159	No
Zinc	0.1658	No
Iron	1.05 × 10^−2^	Yes

**Table 4:** Correlation of fluoride with calcium and pH.

Site	Calcuim	pH	Fluoride Ranges
BH1	0.14	0.68	4.38–6.74
BH2	−0.31	−0.75	5.03–6.40
BH3	−0.94	−0.94	1.70–4.76
BH4	0.06	0.77	1.34–1.66
BH5	−0.55	−0.31	1.17–1.57
BH6	0.12	−0.15	1.46–1.55
BH7	−0.29	−0.04	5.40–5.78
BH8	−0.30	0.04	4.81–5.89
BH9	−0.39	0.11	2.69–4.81
BH10	−0.96	−0.70	0.00–2.79
BH11	0.63	0.34	4.20–5.77

**Table 5:** Correlation of nitrate with chloride and potassium.

Site	Chloride	Potassium	Distance between Pit Latrines and Boreholes
BH1	−0.17	0.64	35
BH2	−0.61	0.01	40
BH3	0.46	−0.68	24, 38, 46 *
BH4	0.99	−0.02	24
BH5	−0.06	0.01	18
BH6	0.67	−0.18	47
BH7	0.99	0.48	34
BH8	0.84	−0.31	27
BH9	−0.36	−0.30	45
BH10	−0.35	−0.42	30
BH11	0.84	−0.31	23.3

* Borehole surrounded by 3 pit latrines.

**Table 6:** Microbial water quality indicators.

Site	Sampling Date	Total Coliform	Heterotrophic Bacteria	*E. coli*	Faecal Coliform
	TWQR	0–5	0–100	0	0
BH12	14 September 2009	211	NM	0	NM
	16 October 2009	0	NM	71	NM
	9 March 2010	121	365	120	22
	7 April 2010	219	370	130	34
BH13	14 September 2009	54	NM	0	NM
	16 October 2009	0	NM	0	NM
	9 March 2010	68	249	21	50
	7 April 2010	50	403	11	44

NM = not measured.
